# Comparison of Intraoperative Doppler Flow Measurements and Algorithmic Flow Estimations in Patients Undergoing Durable Left Ventricular Assist Device Implantation

**DOI:** 10.1111/aor.70150

**Published:** 2026-04-21

**Authors:** Gaik Nersesian, Anna Stegmann, Daniel Lewin, Yuriy Hrytsyna, Leonard Pitts, Julius Kaemmel, Marian Kukucka, Alexander Mladenow, Lisa Garczarek, Julia Stein, Friedrich Kaufmann, Volkmar Falk, Evgenij Potapov, Pia Lanmueller

**Affiliations:** ^1^ Department of Cardiothoracic and Vascular Surgery Deutsches Herzzentrum der Charité (DHZC) Berlin Germany; ^2^ DZHK (German Centre for Cardiovascular Research), partner site Berlin Berlin Germany; ^3^ Charité‐Universitätsmedizin Berlin, Corporate Member of Freie Universität Berlin and Humboldt‐Universität zu Berlin Berlin Germany; ^4^ Department of Cardioanesthesiology and Intensive Care Medicine Deutsches Herzzentrum der Charité (DHZC) Berlin Germany; ^5^ Berlin Institute of Health at Charité ‐ Universitätsmedizin Berlin Berlin Germany; ^6^ Department of Health Sciences and Technology, Institute of Translational Medicine, Translational Cardiovascular Technologies Swiss Federal Institute of Technology (ETH) Zurich Zurich Switzerland

## Abstract

**Introduction:**

Left ventricular assist devices (LVAD) have become an established therapy for end‐stage heart failure over the last three decades. In currently available devices, the blood flow is estimated using a power consumption algorithm. In this study, intraoperative direct LVAD flow measurement with an ultrasonic flow probe was analyzed.

**Methods:**

As part of our routine evaluation of optimal LVAD flow, intraoperative direct flow measurement at the outflow graft was performed using a 14‐mm ultrasonic flow probe. Direct LVAD flow measurements were performed at different speed levels: 4400 rpm, 4800 rpm, 5200 rpm, and the optimal flow setting before chest closure. In twenty consecutive patients who underwent a durable LVAD implantation via median sternotomy in our institution between January and April 2025, direct flow measurement was compared with the estimated flow provided by the device.

**Results:**

Median age was 63 [60;68] years, 17 (85%) were male, 8 (40%) had a history of acute myocardial infarction. Temporary mechanical circulatory support with a microaxial flow pump was necessary in 8 (40%) patients prior to durable LVAD implantation. Destination therapy was indicated in 12 (60%) cases. Mean flows at 4400 rpm were 2.9 ± 0.3 and 3.4 ± 0.4 L/min calculated by the pump and measured by the probe, respectively. Mean difference between the measurements was 0.56 L/min (95% CI [0.40, 0.70], *p* < 0.001). Mean flows at 4800 rpm were 3.5 ± 0.3 and 4.1 ± 0.5 L/min, respectively. Mean difference between the measurements was 0.55 L/min (95% CI [0.33, 0.77], *p* < 0.001). Mean flows at 5200 rpm were 4.2 ± 0.3 and 4.7 ± 0.5 L/min, respectively. Mean difference between the measurements was 0.53 L/min (95% CI [0.40, 0.70], *p* = 0.001). Mean optimal speed for patients was 5400 rpm, mean difference between the measurements was 0.49 L/min (95% CI [0.23, 0.74], *p* = 0.001).

**Conclusion:**

LVAD flow measured with an ultrasonic flow probe shows significantly higher results (approximately +0.5 L/min) compared to the flow estimated by the device. The difference remains constant at different LVAD speed modes. Intraoperative flow measurement employing an ultrasonic flow probe is a valuable tool for hemodynamic evaluation during implantation of a durable LVAD.

## Graphical abstract

Direct intraoperative HeartMate 3 LVAD flow measurements using an ultrasonic probe revealed consistently higher values by approximately 0.5 L/min compared to device‐estimated flows. This discrepancy remained stable across pump speed settings, highlighting the importance of distinguishing measured from estimated flow in HeartMate 3 patients.

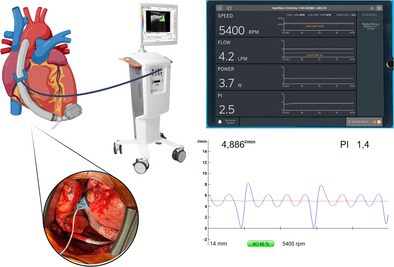

## 1 Introduction

Durable left ventricular assist devices (dLVAD) represent a well‐established treatment for patients with end‐stage heart failure. Durable LVAD implantation can be performed as a bridge to candidacy, recovery, or as destination therapy. The blood flow is one of the clinically relevant parameters, which determine the amount of circulatory support provided by the device. In the currently available devices, flow is estimated using an algorithm which is based on power consumption experiments performed either in ex vivo or in animal models. It presumably exhibits an inaccuracy of up to 10% [1, 2].

Based on echocardiographic and hemodynamic parameters the optimal pump speed is set. A ramp test is performed systematically by increasing or decreasing pump speed [3]. The goal is to achieve maximum pump support while preserving sufficient right ventricular function and aortic valve opening [3].

In our institution, we have applied a modified ramp test for measuring the blood flow in patients undergoing dLVAD implantation. For this purpose, a flexible ultrasonic flow probe was used for intraoperative flow measurements during pump speed adjustments [2]. In the present study, we compare flow values estimated by the dLVAD with those measured by an ultrasonic flow probe.

## 2 Methods

In 20 consecutive patients undergoing HeartMate3 (Abbott Laboratories, Pleasanton, CA, USA) implantation in our institution between January and April 2025 direct LVAD flow measurements using an ultrasonic flow probe were performed during the ramp test for intraoperative LVAD speed adjustments. A 14‐mm ultrasonic flow probe (Medistim, Oslo, Norway) was used and calibrated before each measurement according to the company's recommendations to ensure accuracy. For measurement, the probe was placed around the device outflow graft after LVAD implantation and weaning from cardiopulmonary bypass. LVAD flow measurements and estimated values were simultaneously recorded at different device motor speeds ranging from 4400 to 5600 rpm while achieving device flow outputs of 2.2 to 5.6 L/min. Additional hemodynamic and echocardiographic parameters measured during LVAD speed adjustments were collected.

## 3 Statistical Analysis

Continuous variables were graphically tested for normal distribution using QQ‐Plots and are presented as median (Q1, Q3) or mean (standard deviation), as appropriate. Categorical variables are presented as *n* (%). A one‐sample *t*‐test was used to evaluate the differences between estimated and measured flow values and descriptive *p* values are reported.

Bland–Altman plots were created to demonstrate the flow difference between estimated flows and flows measured by an ultrasonic flow probe vs. mean values between the two measurements. Mean difference between estimated flows and flows measured by an ultrasonic flow probe at different rotational speeds was visualized using box plots.

A scatter plot diagram for estimated flow values vs. flow values measured by an ultrasonic flow probe over a range of device rotational speeds was created. The linear regression function and the root mean square error (RMSE) between estimated flow and direct measurements were calculated.

## 4 Results

Median age was 63 [60;68] years, 17 (85%) were male, and the etiology of heart failure was dilated cardiomyopathy in 10 (50%), ischemic in 9 (35%), and postcardiotomy in 1 (5%) patient. Eight patients (40%) were bridged with a mAFP to a dLVAD (Table 1). Median LVEF was 18 [11; 21], 11 (55%) patients had moderate‐to‐severe mitral regurgitation and 7 (35%) moderate‐to‐severe tricuspid regurgitation (Table 2).

**TABLE 1:** Patient demographics.

Parameter	Values
Age, years^a^	63 [60; 68]
Male sex, *n* (%)	17 (85%)
BMI, kg/m^2^	2.1 ± 0.2
BSA, m^2^	28.5 ± 3.9
History of AMI, *n* (%)	8 (40%)
History of CPR, *n* (%)	3 (15%)
Diabetes mellitus Type II, *n* (%)	8 (40%)
COPD, *n* (%)	3 (15%)
History of CVA, *n* (%)	5 (20%)
Preoperative temporary MCS with mAFP, *n* (%)	8 (40%)
Destination therapy, *n* (%)	12 (60%)

Abbreviations: AMI, acute myocardial infarction; BMI, body mass index; BSA, body surface area; COPD, chronic obstructive pulmonary disease; CPR, cardiopulmonary resuscitation; mAFP, microaxial flow pump; MCS, mechanical circulatory support.

^a^ Median with 25th and 75th quartile, other numerical values as mean and standard deviation.

**TABLE 2:** Patients preoperative echocardiographic data.

Parameter	Values
LVEF, %	18 [11; 21]
LVEDD, mm	71 [64; 80]
LVESD, mm	66 [57; 74]
LVEDV, mL	241[184; 357]
LVESV, mL	176 [160; 291]
CO, l/min	4 [2.5; 4.3]
RVEF, %	40 [27; 46]
RVEDV, mL	186 [93; 241]
RVESV, mL	122 [50; 174]
TAPSE, mm	14 [9; 19]
RVOT VTI, cm	7 [5; 11]

Abbreviations: CO, cardiac output; LVED(S)D, left ventricular enddiastolic(systolic) diameter; LVED(S)V, left ventricular enddiastolic(systolic) volume; LVEF, left ventricular ejection fraction; RVED(S)V, right ventricular enddiastolic(systolic) volume; RVEF, right ventricular ejection fraction; RVOT VTI, right ventricular outflow tract velocity time integral; TAPSE, tricuspid annular plane systolic excursion.

**FIGURE 1:**
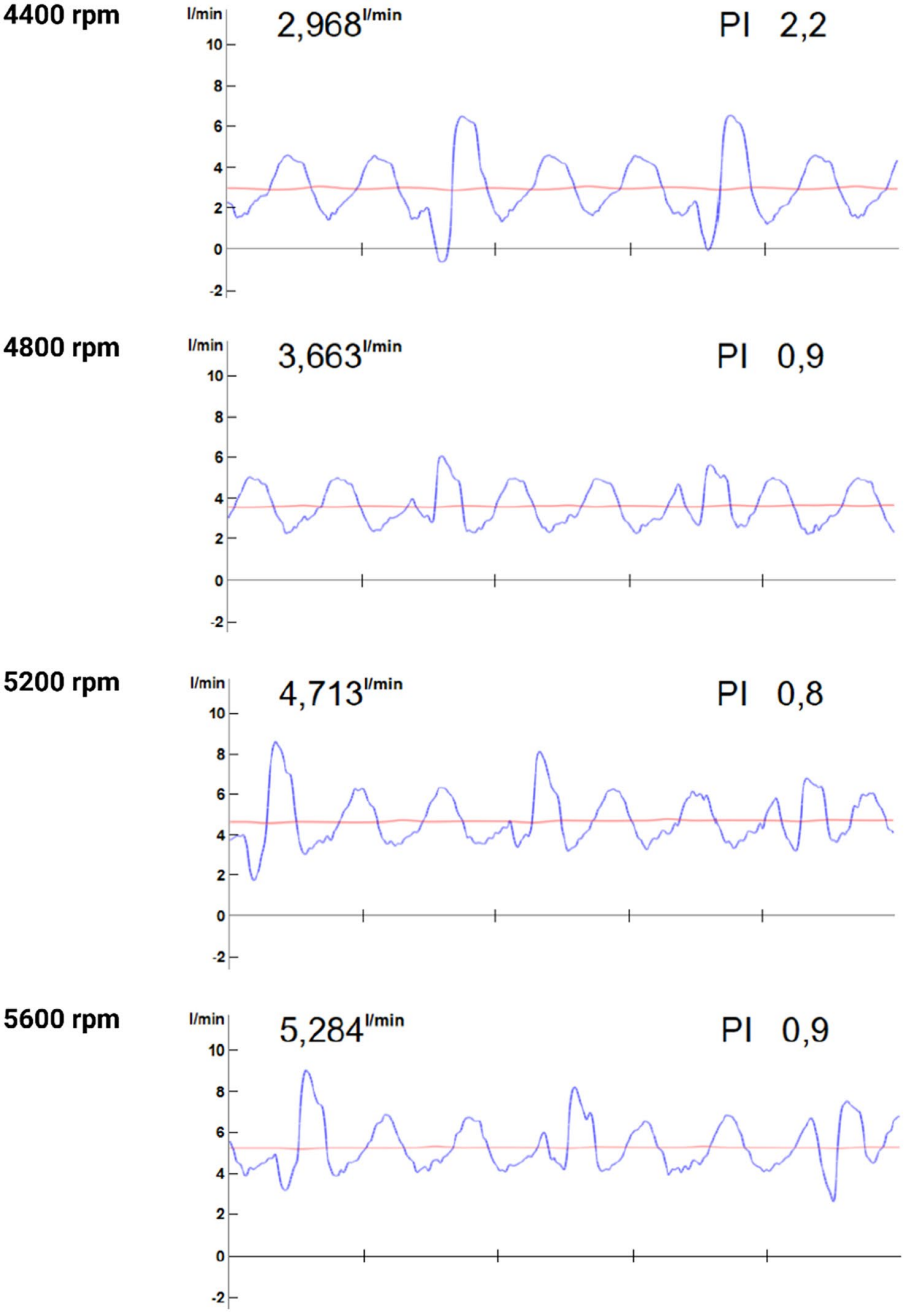
Outflow graft flow waves measured by an ultrasonic flow probe at different speeds. [Color figure can be viewed at wileyonlinelibrary.com]

**FIGURE 2:**
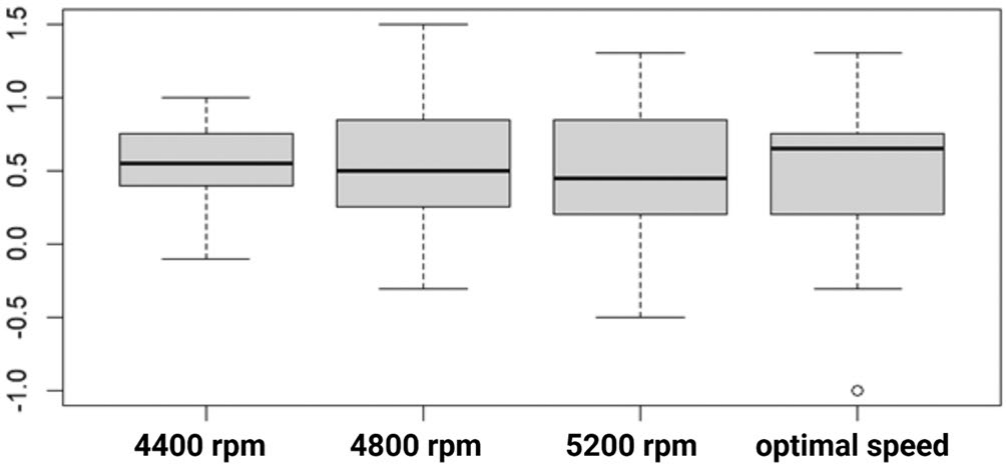
Box‐plots showing average flow difference between the probe and the pump at different rotational speed levels (number of measurements per speed *n* = 20)*. *Optimal speed ranged between 5300 and 5600 rpm, with median speed of 5400 rpm.

Intraoperative dLVAD flow measurements at different pump rotation speeds ranging from 4400 to 5600 rpm were recorded. Flow rates between 2.2 and 5.3 L/min were estimated by the pump, while values ranging from 2.5 to 5.6 L/min were measured by the probe. Samples of HeartMate3 outflow graft waveform recordings measured with the flow probe during low (4400 rpm), medium (4800 rpm), and high (5200/5.600 rpm) output speeds are shown in Figure 1. Comparison of the values has demonstrated that LVAD flow measured with an ultrasonic flow probe shows predominantly higher results (approximately +0.5 L/min) compared to an algorithmically estimated flow by the device (Table 3). This difference remained constant at different speed modes (Table 3, Figure 2).

**TABLE 3:** Summary of flow measurements and differences.

Flow probe (L/min)	Flow pump (L/min)	Mean difference (L/min)	*p*	Power (W)	Pulsatility index	RVOT VTI (cm)
***4400 rpm***						
3.4 ± 0.4	2.9 ± 0.3	0.56 95% CI (0.40, 0.70)	*p* < 0.001	2.8 [2.7; 2.9]	4.6 [3.4; 6.2]	12.8 [10.5; 18.8]
***4800 rpm***						
4.1 ± 0.5	3.5 ± 0.3	0.55 95% CI (0.33, 0.77)	*p* < 0.001	3 [2.9; 3.2]	3.7 [3.3; 4.8]	13 [9.3; 17.2]
***5200 rpm***						
4.7 ± 0.5	4.2 ± 0.3	0.53 95% CI (0.31, 0.74)	*p* = 0.001	3.5 [3.4; 3.6]	3.6 [3.2; 3.9]	14.3 [8.3; 17.5]
***Optimal speed (5300–5600 rpm)***						
4.9 ± 0.6	4.4 ± 0.4	0.49 95% CI (0.23, 0.74)	*p* = 0.001	3.8 [3.6; 3.9]	3.3 [3.1; 3.5]	16.6 [11.7; 24.2]

Abbreviations: RPM, revolutions per minute; RVOT VTI, right ventricular outflow tract velocity time integral.

**FIGURE 3:**
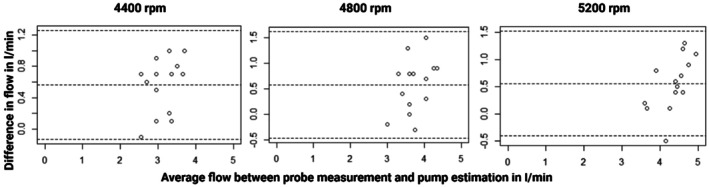
Bland–Altman plots with the flow difference between estimated flows and flows measured by an ultrasonic flow probe vs. mean values between two measurements at different rotational speed levels.

Bland–Altman plots demonstrate the flow difference between estimated flows and flows measured by an ultrasonic flow probe vs. mean values between the two measurements (Figure 3).

**FIGURE 4:**
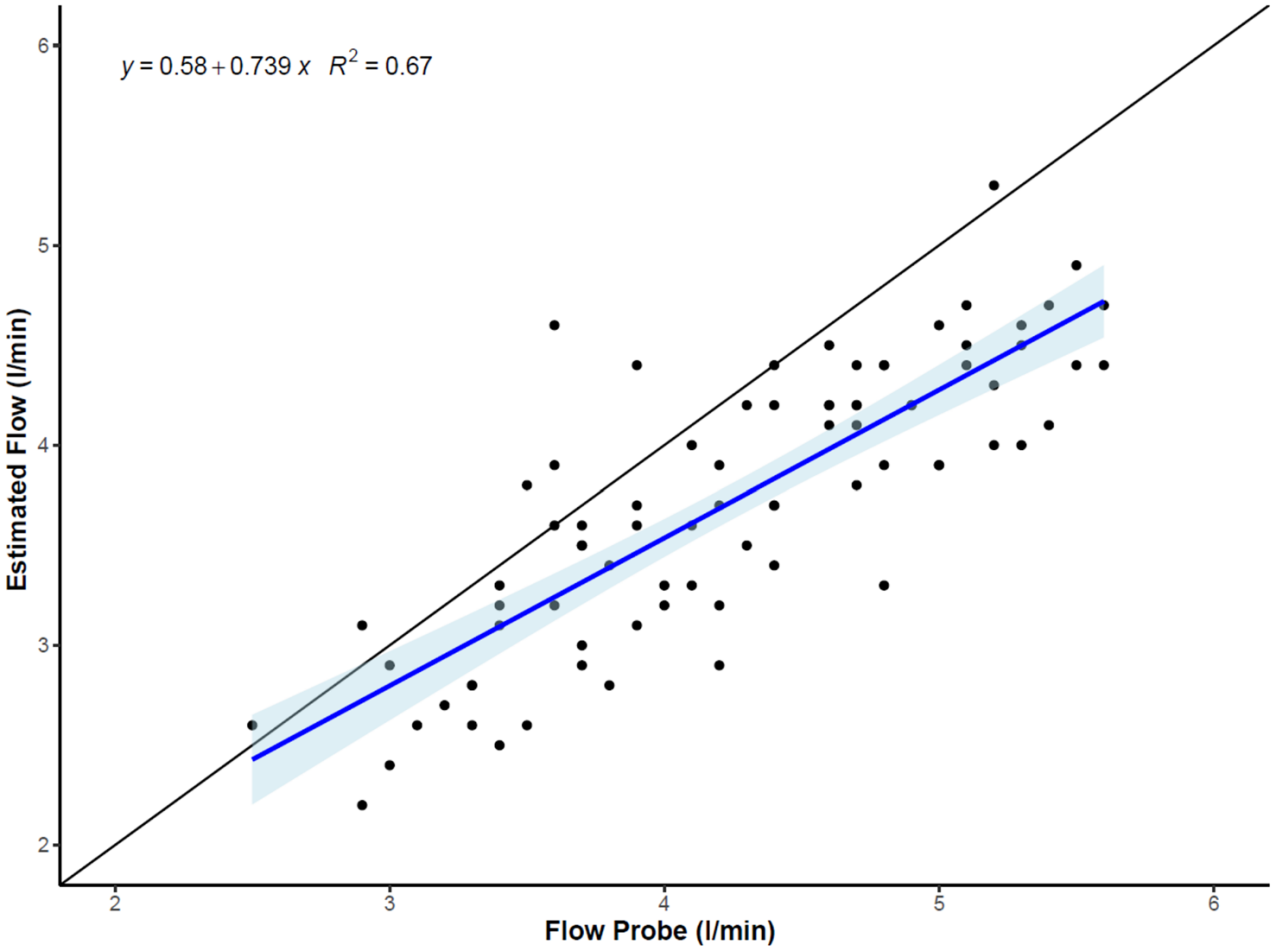
Scatter plot estimated flows and flows measured by an ultrasonic flow probe over a range of device rotational speeds (4400–5600 rpm). [Color figure can be viewed at wileyonlinelibrary.com]

Estimated flows (FE) demonstrated a linear correlation with the ultrasonic flow probe measurements (FP): FE = 0.58 + 0.739*FP, R^2^ = 0.67, root mean square error of 0.69 L/min was observed between flow estimation and direct flow measurement and suggests a difference of 12% to 27% between these 2 measurements at flows of 2.5 to 5.6 L/min (Figure 4).

After dLVAD implantation, 17 (85%) patients were successfully discharged home 3 (15%), to a rehab facility 5 (25%) and 9 (45%). transferred to an external hospital. The remaining 3 (15%) patients developed post‐LVAD right heart failure requiring temporary RVAD support; these patients died in the postoperative course.

## 5 Discussion

To our knowledge, the presented study is the first to report the data on intraoperative flow measurements in patients undergoing HeartMate3 implantation. Our analysis revealed a significant discrepancy between the flow values estimated by the LVAD and the ones measured with an ultrasonic flow probe. A previous study performed in HeartMate II patients demonstrated a discrepancy of 15%–20% between the estimated and measured flows which similarly resulted in an inaccuracy of up to 1 L/min [2].

To date, the only durable implantable LVAD device with an incorporated direct flow measurement was the DeBakey VAD (MicroMed, Houston, TX) which utilized an ultrasonic flow probe (Transonic, Ithaca, NY) attached to the outflow graft. This device is no longer on the market. The currently available HeartMate3 uses an algorithm based on the relationship between power consumption (motor rotor torque, expressed by rotor driver power and rotor speed), and electrical current [4]. This sensorless approach helps to increase the total durability and sustainability of the system over time.

For further clinical decision‐making, it is important to emphasize, that flow data estimated by a HeartMate3 provides valuable trends rather than precise values. At present, the flow estimation algorithm of the HeartMate3 device provides reliable data with an inaccuracy of around ±0.5 L/min, but ranging up to 1 L/min. This is consistent with reported flow underestimation in mock setups. Especially in the high flow region of the HeartMate3 device the pressure‐flow‐characteristic is ambiguous resulting in uncertain and too‐low flow values [5].

In patients with durable BiVAD or dLVAD + temporary RVAD configuration, precise flow data might be more relevant for fine‐tuning and optimization of the balance between the left and the right ventricle. Direct flow measurements might enable improved management of those complex patients. Still, this adaptation would not replace meticulous echocardiographic monitoring to ensure biventricular assessment and adjustment of device settings.

In the postoperative setting, cardiac output (CO) measurements with a pulmonary artery catheter (PAC) represent a valuable tool for hemodynamic monitoring in LVAD patients [6]. Limited data comparing thermodilution‐derived CO values against LVAD flow estimates is available, suggesting a high variability between values measured by PAC and those estimated by LVAD [7]. This discrepancy is explained by the fact that thermodilutionally measured CO may be influenced by multiple factors including body temperature, fluid infusion, and tricuspid regurgitation [7]. However, PAC CO allows assessment of right ventricular function and tracking of trends for patients’ postoperative hemodynamics.

Currently implanted devices are operating with a fixed and constant rotational speed, which precludes the physiological adaptation to patients' circulatory needs. Several concepts for cardiovascular sensing and control have been developed so far. Extravascular hall‐based magnetic flux sensors allow continuous monitoring of cardiac output and blood pressure [8]. Soft strain sensors applied on the surface of the left ventricle allow precise stroke volume and cardiac output measurements [9]. Electrocardiogram‐synchronized pulsatile pump concepts and control algorithms have also been evaluated in ex vivo studies [10, 11]. The rationale for incorporating monitoring sensors and physiological control mechanisms into durable LVADs remains a subject of debate, primarily due to concerns regarding the long‐term durability of such technologies.

Ultrasonic flow measurement is a valuable tool for intraoperative optimization of pump speed in patients undergoing dLVAD implantation. Because the measured flow was approximately 0.5 L/min higher than that estimated by the dLVAD, ultrasonic‐guided speed adjustment may help identify the lowest effective pump speed that provides adequate hemodynamic support while minimizing the risk of right ventricular volume overload.

To demonstrate the advantages of the flow measurement in the intraoperative setting, a new study consisting of two groups—with and w/out intraoperative measurements—should be initiated.

## 6 Limitations

The sample and measurement error of the probe are main limitations of the presented study.

## 7 Conclusion

HeartMate3 flow measurements with an ultrasonic flow probe showed predominantly higher results (approximately +0.5 L/min) compared to the algorithmically estimated flow. This difference remained constant at different LVAD speed modes. Intraoperative Doppler flow measurement is a valuable tool for hemodynamic evaluation during implantation of a durable LVAD.

## Author Contributions

**Gaik Nersesian:** conceptualization, methodology, visualization, writing – original draft, writing – review and editing. **Anna Stegmann:** conceptualization, methodology, visualization, writing – original draft, writing – review and editing. **Daniel Lewin:** writing – review and editing. **Yuriy Hrytsyna:** writing – review and editing. **Leonard Pitts:** writing – review and editing. **Julius Kaemmel:** writing – review and editing. **Marian Kukucka:** methodology, data curation. **Alexander Mladenow:** methodology, data curation. **Lisa Garczarek:** methodology, data curation. **Julia Stein:** methodology, data curation, writing – original draft, writing – review and editing. **Friedrich Kaufmann:** methodology, data curation, writing – original draft, writing – review and editing. **Volkmar Falk:** supervision, writing – original draft, writing – review and editing. **Evgenij Potapov:** conceptualization, methodology, supervision, writing – original draft, writing – review and editing. **Pia Lanmueller:** writing – original draft, writing – review and editing.

## Conflicts of Interest

The authors declare no conflicts of interest.

## Data Availability

The data that support the findings of this study are available on request from the corresponding author. The data are not publicly available due to privacy or ethical restrictions.
